# Singular Phenomena—Reproduction of Enamel, &c., &c.

**Published:** 1859-02

**Authors:** 


For the Dental Reporter.

### SINGULAR PHENOMENA—REPRODUCTION OF THE ENAMEL, &c., &c.

Throughout my researches in the pages of dental history, I have met with nothing that resembles the following curious phenomena. And I trust their apparent incompatibility with received views, will *not* stand as a barrier to the investigation. Whether they will prove to be of practical value, remains yet to be ascertained. But they at least are interesting as physiological facts, and may be the means, not only of casting much light upon the subject, but add also to the literature of Dental Phenomenology. Freely do I confess my inability to assign the causes of these strange freaks of Nature, and therefore submit them to the dental profession, without comment.

CASE 1. Philadelphia, March 12, 1855. Miss S. S., a lady of my acquaintance, aged about twenty years, of sanguine temperament, had, early in life, used a great deal of medicine, the result of which was, the entire destruction of the enamel of all her permanent teeth, while upon the bone or ivory, not the slightest traces of its ravages were visible.

And strange as it may appear, the enamel is again for several years past, being gradually deposited upon them; some of which are now entirely coated over, while others are but half or two-thirds. The formation of this enamel commenced at the *necks* of the teeth, and not at their *cutting* edges, as is usually the case. It is very beautiful in quality, exceedingly white, and of unusual thickness.*

* Some year and a half after the above date, I again visited the city of my nativity—saw my friend Miss S. S., and found the *deposit* still slowly, but gradually progressing.

Case 2. Miss H. L. R. of W——t, New Jersey, aged seventeen, of a healthy appearance, consulted me July 20th, 1856, and presented the anomaly of having no superior lateral incisors. Yet in other respects her teeth were entire and well formed. On inquiry, I ascertained that her grand-mother, previous to marriage, had lost both of her lateral incisors; and that neither the father of H. nor herself have ever had them, either deciduous or permanent.

Wm. W. Morgan.

Somerset, Jan. 3d, 1859.

---

Dover, Del., Nov. 3d, 1858.

Dr. Jno. T. Toland:—Having promised you an article for your *Reporter*, I now furnish you, upon a subject entirely new.

For some time past I have been using the galvanic battery for extracting the "*nerves of teeth without pain*," having originated with myself. The attachment is made in the same way as that in extracting teeth. One pole being applied to the excavator or broach, the other to the patient's hand, and of strength sufficient not to feel too unpleasant, and you can cut away *without the least pain*, and extract the whole of it in a few minutes, without the previous use of arsenic or any other escharotic. In this manner of proceeding no inflammation is set up, to cause suppuration or ulceration at the root. The nerve is cut off like any other part, and after done bleeding, the use of any astringent, (pure Tannin is best) the parts heal, and no danger is apprehended as with arsenic. Thousands of teeth may be saved in this way, that will defy every other method, and that *without pain*. No fear need be entertained of it; you can cut in on the nerve, and no effort will be made by the patient to resist you. The only unpleasantness is that of the battery, which should not be too strong.

My object is to give it to the profession, and if successful, all I will ask, is, to be rewarded by small compensation, instead of securing it by *Patent*.

You will please give this an insertion in your *Reporter*, if not too late, or give, at least, its substance. If too late, report it to the profession verbally from your Depot, and let me hear from it. As to its success no fears are entertained by me, for I know it will do; nor need the practitioner fear to make use of it.

I am the sole originator, and the world is welcome to use it. No patent binds it to any one. Be certain in every case, that all the nerve is gone from the tooth and roots, and I'll guarantee success in every case.

Excuse my haste and oblige

JOHN G. A. BONWILL.

